# ECCE Toolkit: Prototyping Sensor-Based Interaction

**DOI:** 10.3390/s17030438

**Published:** 2017-02-23

**Authors:** Andrea Bellucci, Ignacio Aedo, Paloma Díaz

**Affiliations:** Department of Computer Science, Universidad Carlos III de Madrid, Leganés, 28911 Madrid, Spain; aedo@ia.uc3m.es (I.A.); pdp@inf.uc3m.es (P.D.)

**Keywords:** End-User Programming, toolkits, physical computing, sensor-based interaction

## Abstract

Building and exploring physical user interfaces requires high technical skills and hours of specialized work. The behavior of multiple devices with heterogeneous input/output channels and connectivity has to be programmed in a context where not only the software interface matters, but also the hardware components are critical (e.g., sensors and actuators). Prototyping physical interaction is hindered by the challenges of: (1) programming interactions among physical sensors/actuators and digital interfaces; (2) implementing functionality for different platforms in different programming languages; and (3) building custom electronic-incorporated objects. We present ECCE (Entities, Components, Couplings and Ecosystems), a toolkit for non-programmers that copes with these issues by abstracting from low-level implementations, thus lowering the complexity of prototyping small-scale, sensor-based physical interfaces to support the design process. A user evaluation provides insights and use cases of the kind of applications that can be developed with the toolkit.

## 1. Introduction

Mark Weiser envisioned the real world to be pervaded by a wide range of computational devices that harmonize with the environment, shaping ecosystems made up of coexisting and interweaved devices, interactive surfaces, digital user interfaces, physical objects, sensors and actuators [1]. Such ecosystems, which rely on sensor-based interaction with physical devices, are packed with opportunities to assist and enhance people’s everyday activities. Recent research projects, for instance, have demonstrated how to integrate different sensing technologies in the physical space and take advantage of multi-device interactions to effectively support collaborative tasks such as programming [2] or collaborative learning [3]. However, these projects also reveal that effectively prototyping sensor-based physical interfaces can be a difficult task [4] that, in most cases, requires knowledge of electronics (e.g., build custom hardware with micro controllers), be familiar with hardware drivers, communication protocols and many hours of programming work. The labor of researchers and designers is hindered by challenges (C) that are intrinsic to the heterogeneity of the devices in play and include: 

*C1. Programming interactions with physical components (e.g., sensors, physical inputs and actuators) and digital interfaces (e.g., digital inputs).* Physical systems rely on sensors and actuators to interact with the real world. Sensors convert real world inputs into digital data, while actuators are used to provide physical feedback or actions. Display-enabled devices allow interactions with graphical user interfaces. This means that digital elements can trigger actions in the physical environment through actuators, for instance a digital user interface can be displayed on a smartphone screen to manipulate a pan-zoom-tilt outdoor camera. The same applies the other way round: data from physical sensors can activate digital behaviors (e.g., send me an email when someone opens the door of my room, detected through a motion sensor). Programming the interaction among physical and digital components is not a trivial task because it requires to define mapping strategies and interaction rules for devices with heterogeneous inputs and outputs in order to generate the desired behaviors.

*C2. Cross-device programming platforms and languages.* Devices in an ecosystem vary not only in terms of hardware capabilities but also for development platforms and languages used to program their behavior. Considering, for example, mobile devices and micro-controllers: when the number and type of devices grow, their integration produces a serious development overhead, because expert knowledge is needed in specific programming languages, platforms, Integrated Development Environments (IDEs) or Software Development Kits (SDKs). Solutions that are platform-agnostic (e.g., web-based environments) and that abstract from low-level hardware details are therefore needed to lower the threshold of developing interaction among heterogeneous devices and objects.

*C3. Building custom interactive objects.* Physical objects might be augmented with sensing capabilities and actuators [5], e.g., adding a load sensor to a sofa would allow to notify when someone is seated. This task introduces substantial hardware and software challenges. With respect to the hardware, knowledge of electronics is required to build interactive objects, which in part is made it easier by hardware toolkits such as Arduino [6] or Phidgets [7]. Regarding the software, low-level programming against the particular technology is often necessary: this is a highly specialized task and, most of time, developers cannot reuse the knowledge they acquired with a specific technology when they change to another one.

The first two challenges are related to the need of reducing the effort in programming the user interface and the behavior of multiple interconnected devices, and not just of one device, as addressed by current programming tools. The third challenge addresses the need of building custom devices as part of the ecosystem, as emphasized by the tangible computing research community [8]. Without the appropriate development tools, the implementation of advanced designs is a task dependent on teams of experienced developers. By lowering the skill barrier, toolkits for prototyping complex ecosystems shift implementation efforts from low-level technical details to more sophisticated design nuances [9]. As a result, people without technical skills are empowered to explore and ideate innovative solutions to their quotidian problems.

Even if there are many toolkits already available that ease different aspects of sensor-based interaction, such as tinkering or rapid-prototyping [6], none of them has been designed to specifically support researchers/designers in the integration of physical input/output with other interactive devices, such as smartphones or interactive surfaces, and thus enable to seamlessly trying out different design alternatives. In this paper, we describe Entities, Components, Couplings and Ecosystems (ECCE), a toolkit for assisting researchers and designers with low electronics and programming expertise in the rapid prototyping of small-scale sensor-based physical interfaces as part of their research/design activities. The toolkit aims to lower technological barriers by abstracting from low-level programming through tools for the quick definition of interactions between heterogeneous devices through trigger-action [10] and programming-by-demonstration [11] approaches. While many existing toolkits focus only on physical interaction (e.g., Phidgets [7]) or distributed interfaces and cross-device interactions with existing devices (e.g., Weave [12] or Panelrama [13]), ECCE aims to offer support to untrained end users in the design of custom-built interactive objects using off-the-shelf sensors and actuators. It provides tools for the creation of platform-agnostic web-based interfaces (JavaScript and HTML5) as well as platform-specific code (e.g., Arduino code for custom devices). Finally, it guides the users in the deployment of the system logic into the actual devices of the ecosystem. The toolkit, therefore, represents an advance of the state of the art with respect to the ease of programming both the software and the hardware side of systems that interweave physical and digital objects. This research advances our previous work [14,15] and provides novel contributions to the body of knowledge on sensor-based interaction by (i) exposing the complexities of developing small-scale physical systems; (ii) providing a survey of the state of the art; (iii) an in-depth discussion of the design goals pursued by the ECCE toolkit; (iv) emerging designs from two workshops (8 and 16 participants) that shed light on the usefulness of the toolkit as well as on what functionality are needed for an end-user toolkit to support novice users during the design process.

The remainder of the article is structured as follows: first, the contribution is framed with respect to the state of the art. Then, the ECCE toolkit is presented, focusing on the challenges that led to the design of functionality to support the design process. We then present results from two workshops we conducted to evaluate the toolkit range and support to prototyping activities and to highlight the strengths and limitations of this research. Finally, we draw conclusions and outline the road ahead for future development.

## 2. Related Work

We discuss the state of the art of toolkits for sensor-based physical computing and cross-device interactions. We also examine the literature on End-User Programming, focusing on techniques to allow non-expert users to create physical interfaces. The goal of ECCE is to support the design process through rapid prototyping by easing the programming of physical/digital interactions: it is therefore out of the scope of this research to address infrastructure issues for the development of medium to large scale ecosystems (e.g., HomeOS [16] and OpenHAB [17]). We frame our contribution according to the three aforementioned challenges (see Table 1 for an overview):

1. Physical/digital interactions: does the toolkit support the development of applications that combine physical input/output with other platforms, such as smartphones, tablets or interactive surfaces?
2. Cross-device programming: does the toolkit simplify development by a common programming environment and language for different platforms or devices?
3. Build custom interactive objects: does the toolkit offer support to the end user in building custom sensor-based physical devices?

Earlier evaluations of physical computing environments have been carried out [18,19] that uncover the problems end users encounter when developing physical systems as well as the functionality an end-user toolkit should expose to support untrained users in this kind of programming task. In particular, the study from Booth et al. [19] showed that, to be successful, users are expected to be sufficiently proficient at both programming and building electronic circuits. The knowledge of electronics required to build interactive objects is in part made it easier by the availability of hardware toolkits [6,7]. Regarding the software, low-level programming against a particular technology is a highly specialized task and developers have been found to struggle with debugging and program construction. Other works [20] have outlined learning barriers end-users face in programming Arduino micro-controllers with a textual and visual development environments. Issues span from design barriers, for instance the user does not know if a sensor has to be connected as an input or output, to understanding barriers, which occurs when users were not able to understand “why something did not happened when it was supposed to” [20]. The study suggests that visual environments have a positive impact on the user experience and that visual languages are a promising tool for supporting untrained end users in physical programming tasks.

### 2.1. Toolkits for Physical Computing

Arduino [6] was developed to ease the creation of physical interaction. Arduino is an open-source electronics prototyping platform based on flexible, easy-to-use hardware and software. It still requires electronics and programming knowledge. Integration of sensors and actuators into a prototype, in fact, requires the building of a circuit, which connects the sensors with the Arduino micro-controller, and programming the software of the microcontroller to make sense of the data gathered. Arduino IDE, moreover, offers basic functionality for programming and compiling the source code and lacks the features of modern IDEs, such as automatic error detection and code completion. Phidgets [7] and iStuff [21] are other toolkits that ease physical prototyping. Phidgets works on the level of electrical components by providing an extensive range of ready-to-use physical input devices, such as buttons or sliders, sensors such as touch or proximity and actuators such as servo motors or relays. It offers an Application Programming Interface to define the behavior of physical widgets, thus targeting users with programming expertise. iStuff does not have the versatility of the two previous toolkits in terms of combinations of physical inputs/outputs but its design rationale and software architecture focus on prototyping interactive physical environments. iStuff, in fact, provides a platform to connect lightweight devices that enable users to interact with displays and user interface applications that coexist in a digitally-augmented space. 

### 2.2. Toolkits for Cross-Device Interactions

Given the increasing availability of multi-surface environments, many high-level tools have been proposed for building applications that support distributed user interfaces that span across multiple surfaces and devices [12,13,22,23]. The majority of tools use web-based technologies to build device- and platform-agnostic interfaces. However, current solutions (i) focus only on off-the-shelf devices and do not provide support for novel sensor-based interaction with custom-made interactive objects; and (ii) they heavily rely on textual programming (e.g., scripting languages), thus targeting tech-savvy users. Weave [12], provides an authoring environment for interweaving off-the-shelf wearables and mobile devices. It uses JavaScript for the definition of cross-device behaviors and HTML for the user interface components. Panelrama [13] targets web applications and enables cross-device interaction by extending the HTML language with additional tags for the definition of distributed interfaces. XDStudio [22] provides a visual tool for interactively designing cross-device interfaces. It facilitates the simulation of target devices, thus enabling the authoring of cross-device behaviors on a single device, and also the deployment of the generated interfaces on the devices. WatchCONNECT [23] explores sensor-based interactions focusing on smartwatches. It represents an exception to the previous toolkits and APIs since it provides a custom and extendable platform for prototyping smartwatches interactions with other off-the-shelf devices. However, the toolkit focuses on gestural interaction with wearable devices and does not support, for example, the implementation of other types of custom tangibles. It also seems to support only interactions between one single wearable device and another display-enabled device.

### 2.3. End-User Programming for Ubiquitous Interaction

As reported by Lieberman et al. [24], techniques to support end users in the programming task include: scripting languages, domain-specific languages, programming-by-demonstration, tailoring, configurability, visual languages and natural programming environments. Relevant for this research is visual programming, which allow end-users to create programs by manipulating graphic elements rather than by textual specification [25] and programming-by-demonstration, in which “users provide example interactions and the system infers a routine from them” without requiring textual programming [24] (p. 3).

d.tools [26] is one of the first attempts to introduce End-User Programming (EUP) techniques for authoring ubiquitous interaction. It supports visual programming via a visual state chart editor. The system was later extended by Exemplar [11], which exploits the programming-by-demonstration technique to enable interaction via sensor data. iStuff mobile [27] is built on top of iStuff [21] and it provides a software architecture and visual language to prototype interactions between physical objects, enhanced with sensors and actuators, and mobile devices. The project pushes forward the vision of interweaved devices, but no information is provided about (i) the implementation effort required to program cross-device interactions; (ii) how the system bridges communication among heterogeneous devices; or (iii) how the system could be extended to include new devices. Other environments have embraced visual programming for prototyping physical interaction. For instance, Scratch4Arduino [28] exploits the Scratch visual environment and visual syntax [29] to program the Arduino hardware through the composition of logical blocks on the screen. However, Scratch4Arduino is meant for educational purposes and to ease the transition to a classical textual programming language, thus it does not provide support for building complex device ecosystems. More advanced tools for this task are Node-RED [30] and MIT App Inventor [31]. Node-RED implements a visual data-flow language to interweave smart things. It provides high configurability and extensibility and it is powered by crowd-based development that enables people to reuse code created by others. However, users still need to have programming knowledge to create useful programs, it does not support interactions with mobile devices and does not offer direct support for interconnecting devices. MIT App Inventor offers a visual environment for building mobile user interfaces via drag-and-drop graphical elements and programming device behaviors (including sensors) via a Scratch-like approach. It is limited to mobile devices and it does not support cross-device interfaces. Therefore, integrating different devices in the same environment would still require considerable engineering effort.

## 3. The ECCE Toolkit

To address the previously mentioned challenges, the ECCE (Entities, Components, Couplings and Ecosystems) Toolkit pursues the following design goals that has been informed by the analysis of the state of the art, with a special focus on end-user development for physical computing [19,20], and our personal experience in developing physical systems in our research laboratory (e.g., [32]): 

*G1 Support physical design*: Prevent users’ error in circuit construction [19] by providing tools that support the end user both in the circuit design as well as the physical assemblage of electronic-incorporated objects.

*G2 Span heterogeneous hardware*: Support the seamless inclusion of diverse off-the-shelf devices (e.g., smartphones and tablets) as well as hardware platforms for building custom electronic-incorporated objects (e.g., low-cost microcontrollers). 

*G3 Extensibility*: Extending the API both at the micro-level (adding new functionality within the context of a single project) as well as at the macro-level (extending the toolkit for the entire end-user community by adding new devices and functionality).

*G4 Ease of physical/digital programming*: Support end users in creating interactions between physical and digital component with end-user development tools and techniques that ease program construction for physical interaction. As the state of the art uncovers [19], in physical computing tasks users struggle with basic programming activities, such as read data from sensors or define the correct threshold values for triggering sensor-based events.

The state of the art shows that EUD is critical for the development of device ecosystems, since it is inconceivable to determine at design time all the possible configurations of ubiquitous technologies and the way users are willing to interact with them. There is also a clear evidence that coping with the integration of heterogeneous technologies is a difficult task (G2) [4]. The technical complexity of building device ecosystems limits their current design [7]: as a result, high level activities are often inhibited by simplistic implementations. We cannot assume a high level of technical expertise for the designers using ECCE. Supporting unexperienced users (G4) aims to include new design participants [33] and favors faster adoption of the toolkit, which in turn would provide opportunities to better understand the needs of designers/researchers working on the development of physical computing systems. The main goal of the ECCE toolkit is to offer tools to bypass the technological barrier, hence making the setup of device ecosystem less burdensome and allow end users to focus on their primary task. Our toolkit builds on the top of previous research and adds EUD support in terms of a comprehensive visual environment that allows to connect off-the-shelf mobile devices with custom-built physical interfaces (G2), assisting the physical design (G1) as well as the implementation and deployment of augmented devices together with the definition of interactions among them (G4). 

**Figure 1:**
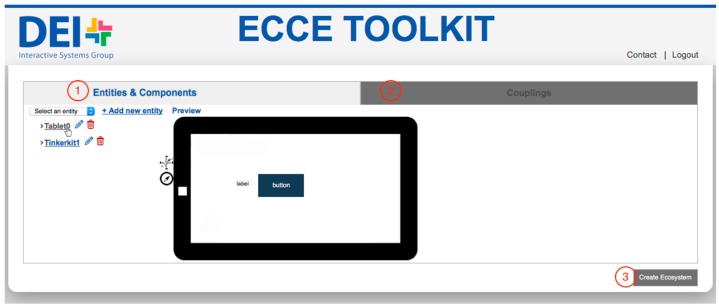
Web interface of the ECCE Authoring Environment to create an ecosystem: (1) add/edit new entities via the Entity & Components Editor and (2) define their behavior (Couplings Editor). By selecting an existing entity, a preview gives users a prompt feedback regarding the entity design. (3) Users can automatically generate the runtime code of the entities as well as the server logic (Ecosystem Code Generator).

**Figure 2:**
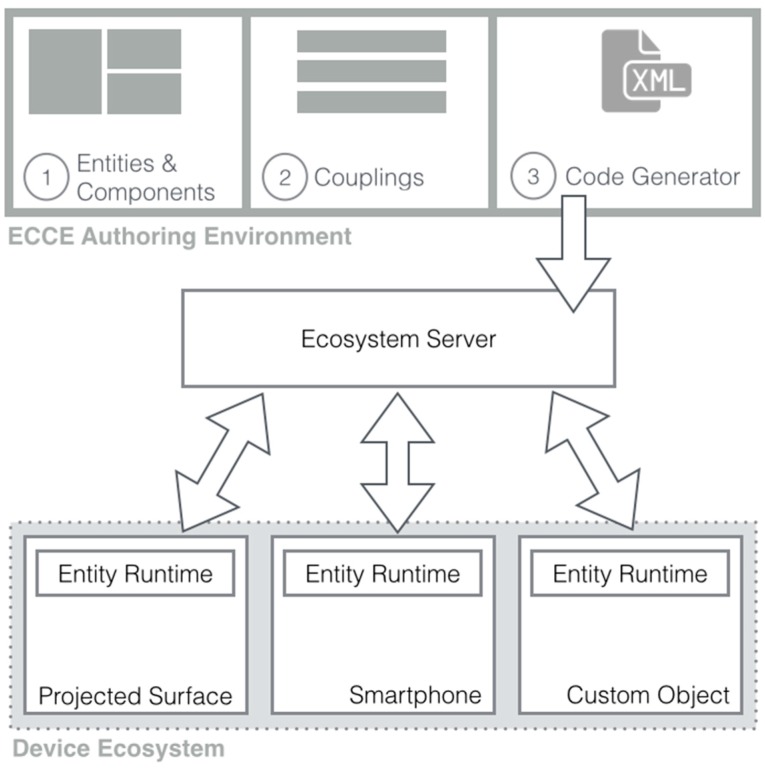
The architecture for creating a device ecosystem with ECCE.

The toolkit implements a graphical web-based interface for authoring sensor-based physical interactions (Figure 1). The ECCE Authoring Environment provides three main modules (Figure 2):

1. The *Entities & Components Editor* assists the design of the user interface as well as the physical assemblage of sensor and actuators. ECCE supports both readily available devices such as smartphones, tablets or interactive tabletops and surfaces as well as electronic-incorporated objects, e.g., Arduino-based physical user interfaces. This module addresses challenge C3, (building custom sensor-based electronic-incorporated devices) through design goals G1 (support physical design) and G2 (span heterogeneous hardware);
2. The *Couplings Editor* enables the user to define interactions between devices by means of event-based behaviors, taking into account the capabilities of the components of each device. This module enables interaction with physical and digital elements (C1) adhering to design goals G2 (span heterogeneous hardware) and G4 (ease of physical/digital programming). It enables end users to define interaction rules and sensor trigger values by means of a graphical interface and exploit the programming-by-demonstration paradigm, thus coping with aforementioned issues of program construction for physical computing (G4).
3. The *Ecosystem Code Generator* automatically generates the *Entity Runtime* source code (web applications as well as microcontrollers code) by parsing XML-descriptors created with the authoring tool. The runtime code is deployed on target devices according to its development environment and capabilities, thus addressing C2 (managing different platforms and programming languages). The *Ecosystem Code Generator* also instantiates the logic of the *Ecosystem Server* that embeds the functionality to setup and to transparently manage data routing among heterogeneous networked devices and sensors (G2).

ECCE has been designed to provide a modular architecture based on definition of devices through XML templates and to allow designers and developers to extend the features for a specific project or an entire community (G3). The user interface of the authoring environment follows the rationale of similar environments such as MIT App Inventor [31]; it provides editors that conceptualize the workflow into separate tasks of (i) building the interface; (ii) programming the behavior and (iii) deploy and run the application. 

### 3.1. Composition of Interactive Devices with the ECCE Authoring Environment

**Figure 3:**
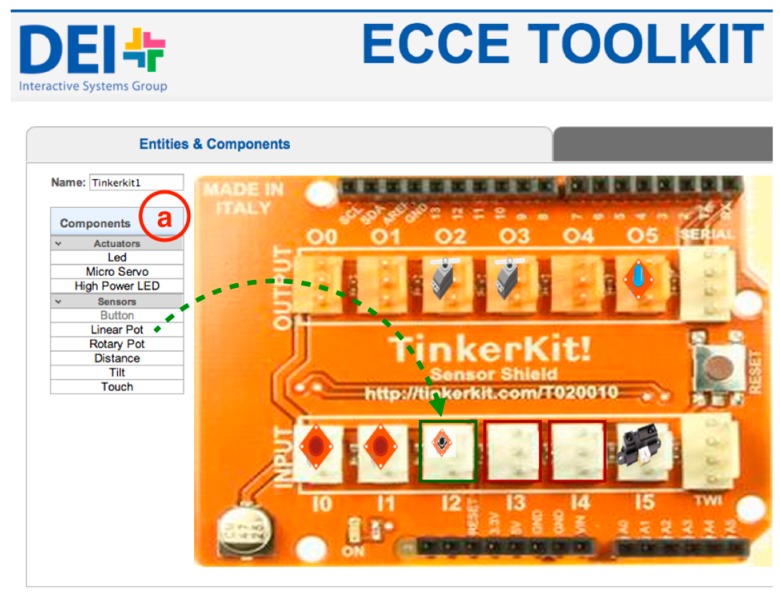
A screenshot of the web interface for the definition of Tinkerkit-based interactive entities. Users can (**a**) drag-and-drop sensors and actuators from a palette of components to the desired port.

**Figure 4:**
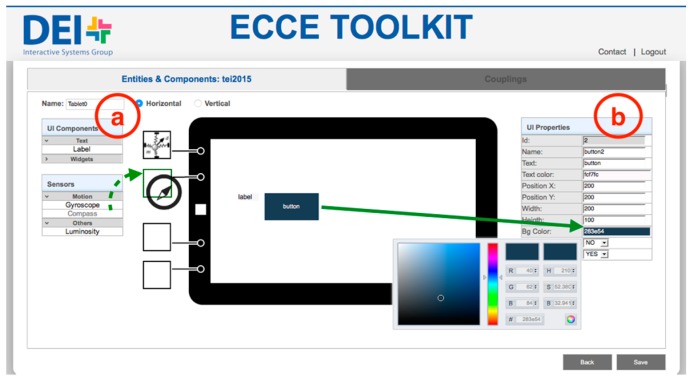
A screenshot of the web interface for editing off-the-shelf devices with a display screen. User can (**a**) add sensors and interface elements from a palette, and (**b**) configure the properties of digital elements on the screen.

The *Entities & Components Editor* (Figure 3 and Figure 4) module enables new devices to be added to the ecosystem by (i) using existing mobile devices such as tablets or smartphones, laptops and multi-touch surfaces such as tabletops, see-through displays or projected surfaces or (ii) building custom sensor-based interactive objects with off-the-shelf micro-controllers, sensors and actuators. In the Entities & Components Editor, each entity is designed as the aggregation of different components both physical and digital. Examples of physical components that are (i) sensors such as accelerometers, gyroscopes, distance, luminosity, load and flex sensors; (ii) physical input devices such as potentiometers, joysticks or RFID readers; and (iii) actuators such as speakers, motors or LEDs. Digital components—the elements of the graphical interface—can be defined for entities that feature a display screen. They are labels, digital buttons, sliders, video streams and the like. New entities can be created by selecting from a list of predefined entities (Figure 1). 

Once the user chooses to add a new entity or edit an existing one (Figure 1), the web interface allows to configure entities by dragging-and-dropping elements from a palette (Figure 3a and Figure 4a). The web interface generates an XML description of the interactive entity from templates of display-enabled devices or micro-controller based interactive objects. Such description will be parsed by the *Ecosystem Code Generator* to generate the software logic to manage physical and digital input/output on host devices (the *Entity Runtime*). This approach provides the backbone for the development of physical/digital interactions that are independent of the underlying hardware and can be extended to include additional elements (Section 3.5). An XML file describes both the physical and the digital components of an interactive object, and provides the references for the definition of interaction rules between physical and digital elements [15]. 

**Figure 5:**
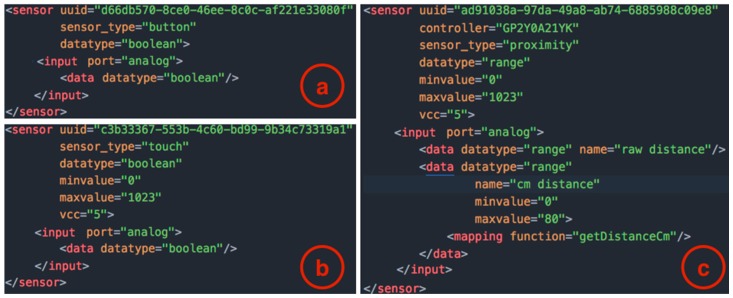
Excerpts from the XML file with the templates for the definition of sensors: (**a**) a button; (**b**) a touch sensor and; (**c**) a proximity sensor.

As an example, Figure 5 shows excerpts from the XML file that stores the templates for physical sensors in the ECCE toolkit. Each sensor has a field (*sensor_type*) that defines the type of sensor (e.g., button, touch, proximity), an optional *controller* that identifies different implementations of the same sensor type (e.g., infrared proximity sensor versus ultrasonic range finder) and a unique identifier (*uuid*). Each sensor generates data of a different *datatype*—(i) high/low *boolean* values; (ii) discrete values within a *range* or; (iii) *string*—that can be mapped to produce output values according to different mapping functions. For example, a proximity sensor can send raw values as directly read from the sensor or use a utility function (*getDistanceCm* in the example in Figure 5) to convert raw data in the actual distance in centimeters. The XML code in Figure 5 is provided by the toolkit and it can be extended by users to create additional functionality or add new sensors as shown in Section 3.5. The XML description provides templates for sensors and UI elements that are completed with additional information at the time the user selects the component in his/her project. For example, the actual *pin* for a sensor will be specified in the *output* tag once the user will drop the sensor in the desired port (Figure 3).

For interactive objects, the toolkit visually supports the physical design by providing an interface that resembles the physical board and guides the users to plug the desired sensors and actuators in the correct port (Figure 3). The physical board provides a color code—white for inputs components and orange for outputs components—and the interface allows to drop components only in the correct port, thus supporting users in avoiding configuration errors. In the case of available devices, the toolkit grants access to built-in sensors (e.g., triple-axis accelerometer, magnetometer and light sensor for Android devices) and supports the design of the graphical user interface (Figure 4). Users can drag-and-drop digital components on the representation of the display screen (Figure 4a)—proportional to the real screen size—and customize the look-and-feel and properties of each component (Figure 4b).

### 3.2. Definition of Cross-Device Behaviors

**Figure 6:**
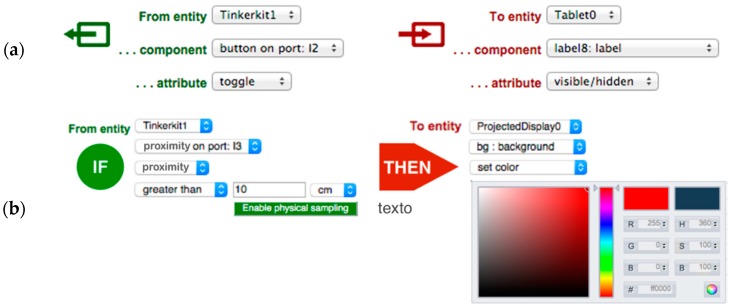
Screenshot of the interface for configuring cross-device behaviors. (**a**) A direct mapping: The button on port I2 (see Figure 3) is used to toggle the visibility of a label on the graphical interface of a tablet; (**b**) A trigger-action: If I am at more than 10 cm from the distance sensor change the background of the projected display to “red”.

The previous tool provides a coherent definition of all the objects in the ecosystem in terms of their components and attributes. The *Couplings Editor* exploits these descriptions to link components using rules that manage the interplay between physical and digital components. Again, a graphical interface is provided for the end-user configurability of behaviors (Figure 6). ECCE has been developed to allow a wide range of integration of physical and digital components following an event-driven approach. At this stage of development, cross-device behaviors can be implemented with two sensors/actuators couplings:

- *Direct mappings*: there is a one-to-one mapping of the input value into the outcome value. The toolkit manages the mapping for the user and the interface enables users to select only viable mappings (e.g., Boolean input values with boolean outputs, discrete input values with discrete outputs, etc.). An example of a direct mapping is linking the movement of a potentiometer to the rotation of a servomotor. The potentiometer provides values from the range *0..1023* and, therefore, a mapping from *0..1023* to *0..179* will give the correct output for the servomotor. On the target object, the actuator will update its status according to the output value;
- *Trigger-action rules*: “if-this-then-that” productions, which have demonstrated to be powerful enough to enable a wide range of smart behaviors for device ecosystems [10]: if the value of the event matches the trigger of the rule, then the corresponding output is activated on the target object. An example is changing the color of a digital object (e.g., the background of a projected display) when someone in the physical world is far from a particular physical object augmented with a distance sensor (e.g., a door).

### 3.3. Programming-by-Demonstration

**Figure 7:**
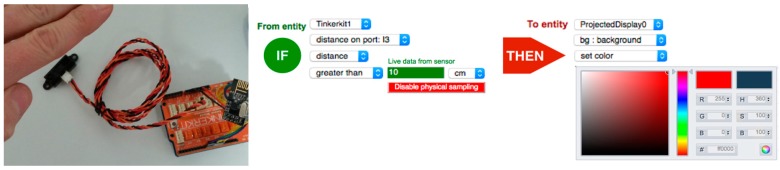
Programming-by-demonstration. The user interacts with the sensor to complete trigger-action rules with live sensor data.

To assist users in the definition of trigger-action rules, the toolkit implements programming-by-demonstration (PBD) functionality [11]. By installing the ECCE PBD firmware on a target device, this will act as a sampling unit for sensor data corresponding to the current entity design as defined with the *Entities & Components Editor*. In this way users can configure trigger-action rules by completing the rule with actual sensor readings, thus using the physical environment as an interface for programming. For instance, in the case of the second rule in Figure 6, instead of having to type the distance, the user can simply show the distance to the sensor, which will provide the corresponding data (see Figure 7).

### 3.4. Running the Ecosystem

**Figure 8:**
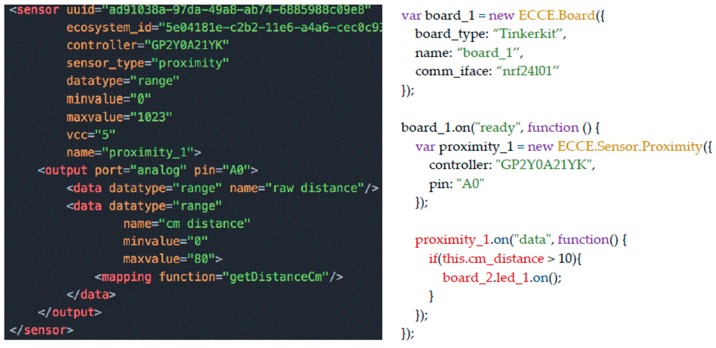
An example of the JavaScript code on the server (on the **right**) that is generated by parsing the XML definition of a proximity sensor (on the **left**).

The interface of the authoring tool provides a button to run and test the current ecosystem configuration, which will launch an instance of the *Ecosystem Server*. All the XML-based descriptions are parsed by the *Ecosystem Code Generator*, which creates the logic of the *Ecosystem Server* in terms of data structures that hold the description of interactive objects, their interactions and network-agnostic data routing. As an example, once the XML description in Figure 8, on the left, is parsed, the JavaScript code in the same Figure, on the right, is generated that allows the *Ecosystem Server* to manage events from the Entity that holds such sensor; in this case it will turn on the LED on another entity if the distance is lesser than 10 cm. The *Ecosystem Code Generator* also generates the source code source code for the user interface of display-enabled objects (a HTML5-CSS-JavaScript webpage), which can be executed by scanning an auto-generated QR-Code. This avoids writing the URL of the generated interface on the target device.

**Figure 9:**
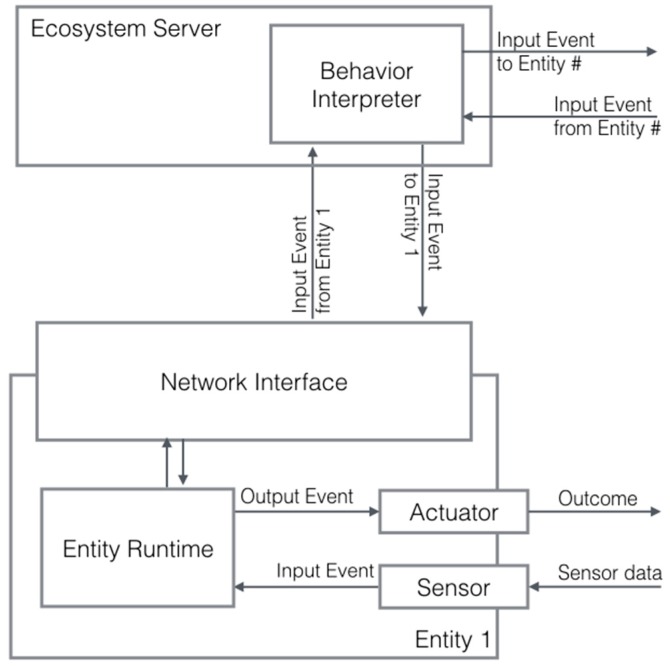
The Entity Runtime. Input events from sensor data and remote events from other entities are interpreted (Behavior Interpreter) and the corresponding output event to another remote endpoint (or to the same entity as well) is generated.

The *Ecosystem Server* acts as a central communication unit. All the messages from one object to another pass through it and it maintains the data structures for validating the interaction rules between physical and virtual objects. As implemented by the *Entity Runtime* (see Figure 9), the server receives events from remote sensors and dispatch the event to the corresponding entity. On the server side, the *Behavior Interpreter* checks if the event meets a specific trigger value defined in the interaction rules and, if there is any, it generates a message with the corresponding outcome to be routed to other devices (including the source device) that (i) will activate an actuator or (ii) will update an attribute of a digital element on a display-enabled device, according to the received output event. 

The server also bridges heterogeneous networking interfaces. It stores a lookup table of all the objects and their networking interfaces so to provide a network-independent routing mechanism. Users do not need to know or to program the communication between different networked objects: the server automatically handles all the connections transparently thus abstracting from low-level communication protocols. 

### 3.5. Further Implementation Details

The current version of the toolkit has been implemented in Node.js [34]. The server is built upon the Express Web framework [35]. It uses a combination of HTML5, JavaScript, and CSS to generate the logic and the user interface for display-enabled entities and the Johnny-five JavaScript platform [36] to manage Arduino-based devices. 

The toolkit supports different platforms for creating cross-device interactions. Being the user interface for display-enabled devices built on top of web technologies, it can be deployed in any modern browser thus supporting desktop (Windows, Mac OS and Linux) as well as mobile operating systems (Android, iOS and Windows 10 Mobile). The user interface is designed and deployed on the host device according to the display features such as dimensions and resolution. To ease the development, the toolkit provides a set of predefined display-enabled devices: desktop or laptop screens of different sizes and resolutions (ranging from 13 inches at 1280 × 800 pixels to 29 inches at 2560 × 1080) and a range of mobile devices such as Samsung Galaxy S4/S5/S6/S7, Samsung Galaxy Note 10, Google Nexus 4/5/6/7, iPhone 4/5/6/6+ and the iPad family. Users can also add custom devices by defining a device name, display dimensions, pixel ratio, available sensors and the type of platform (either mobile or desktop). With respect to micro-controllers, the current implementation supports the design of custom objects building on top of Arduino-based micro-controllers and the Tinkerkit hardware toolkit [37]. The *Entity Runtime* for micro-controllers implements a custom version of the Firmata protocol [38] to support Inter-Integrated circuit (I2C) communication. Available sensors, input devices and actuators are listed in Table 2: The ECCE toolkit support Arduino-compatible 3-pins components (ground, 5V, signal) from a wide range of do-it-yourself electronic manufacturer, such as Sparkfun [39], as well as commercial sensors kits (e.g., Duinotech 37-in-1 sensor kit [40]). The communication with display-enabled entities occurs via WiFi and it makes use of WebSockets. For electronic-incorporated objects, the toolkit supports Serial, nRF24L01+ Ultra Low Power 2.4Ghz Radio Frequency [41] and Bluetooth Low Energy communications.

### 3.6. Adding New Sensors/Actuators to the Toolkit

As discussed in Section 3.1, users can exploit the XML definition of sensors, actuators and user interface elements to extend the functionality of the toolkit by adding new components or new mappings for the sensors data. Taking as an example the definition of the proximity sensor in Figure 5, users could extend the sensor functionality by adding a new data field and write a function that transform the raw data into distances in inches. The new attribute will be then available to be used, as shown in Figure 8 for the distances in centimeters. Users can add new sensors/actuators to the platform by encapsulating components already supported by the Johnny-Five library into ECCE toolkit components, which is achieved by extending the *ECCE.Sensor* class that encloses the logic to define abstract sensors’ features and attributes (e.g., their names, ids, controllers, etc.) as defined in the templates. This mechanism, which relies on the Firmata protocol, makes it easy for users with limited technical skills to add new sensors to the platform. However, the design choice limits the capabilities of the framework to the communication capabilities of Firmata. For instance, at the present time, it is not possible to include RFID/NFC readers because they rely on Serial Peripheral Interface (SPI) communication, which is not currently supported by Firmata. In this case, extending the toolkit functionality would be readily possible for end users by creating a custom script to be uploaded on the micro-controller which would replace the Firmata functionality for the specialized function (e.g., read RFID tags). The script would write data according to the selected network interface once the RFID has been discovered and a general purpose ECCE object would read for data from the micro-controller.

**Figure 10:**
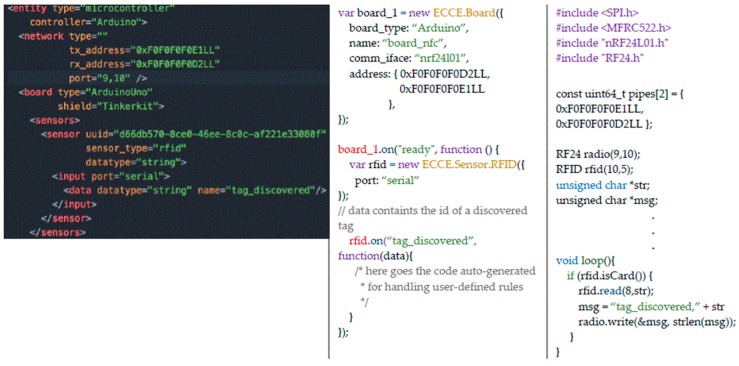
An example of the code to extend the toolkit’s functionality and include a RFID reader input device. On the right, the XML descriptor of an entity with a RFID reader. In the middle, the JavaScript code that is generated by parsing the XML descriptor. On the right, the custom Arduino code for the RFID reader.

Figure 10 shows an example of this scenario for the RFID RC522 module. The toolkit provides a template for a generic sensor that the user has extended to create an *ECCE.Sensor.RFID* sensor that sends messages over nrf24l01. To this end, the user first has had to create the XML template for the sensor (Figure 10, on the left) and then the JavaScript code that is generated once the XML is parsed. The transmitting and receiving address of the nrf24l01 communication interface are automatically generated by the toolkit when the user adds the new device (Figure 1). The *tag_discovered* input event is registered by the *ECCE.Board* event listener that, in this case, will listen for *tag_discovered* events over nrf24l01 (Figure 10, in the middle) and will fill in the code to handle the callback according to the interaction rules previously defined. Figure 10, on the right, shows the Arduino code the user has to write to implement the functionality to send *tag_discovered* events. Since this task requires specific programming knowledge, the toolkit provides a library of predefined sample sketches that users could use and tailor according to their needs. In this case, sketches are provided that setup basic nRF24L01 communication and the user only has to insert the correct address as provided by the toolkit and write the loop functionality to send the message for the *tag_discovered* event type.

## 4. User Study

The contribution offered by the development of a new toolkit to technical HCI research is indirect in nature and is framed under the concept of enabling research [42]: the researcher pursues the goal to empower others to “address a need by making it possible, easier, or less expensive for future inventive work to do so” [42] (p. 74). Performing a formal evaluation of a toolkit is impractical [33] and, while usability metrics have been successfully applied to evaluate interactive applications, there is no established technique to evaluate the toolkits that support the development of such applications [43]. Myers et al. [44] propose five “themes” for the evaluation of toolkits that stem from an analysis of successes and failure in the history of user interface software tools. Evidence from the state of the art [4,21,45] shows that the evaluation of a new toolkit for the development of ubiquitous environments capitalizes on use cases to demonstrate how the toolkit allows to build relevant applications while it eases the programming efforts. According to Myers et al. [44] definition, this is described by the tradeoff between the *threshold,* or the capacity of lowering the skill barriers, and the *ceiling,* or the capacity of enabling meaningful interactions. To investigate the *threshold/ceiling* of the toolkit, we opted to run two workshops that would uncover the applications that could be constructed with ECCE, the ease of programming and the qualitative differences in terms of programming effort by expert and non-expert participants. We acknowledge that the chosen evaluation format prevents from reaching reliable conclusions on the effectiveness of the toolkit. 

Data was collected as both in video and personal observations during the workshops as well as oral group interview at the end of the workshops. The results are presented as overall findings on the use of the tool and examples of individual projects that were developed during the workshops. We postpone empirical evidence for future research.

### 4.1. Workshops Overview

Both workshops consisted of two main phases:

1. *A design task*, in which participants discussed and came up with design ideas of a device ecosystem for a specific context and,
2. *An implementation task*, in which participants used the ECCE toolkit to implement their design ideas.

We framed the design task in the context of cultural heritage: participants would be designing a digitally-augmented smart exhibit about the Mayan culture. The team were given sheets with a description of some peculiar objects of the Mayan culture together with 3D-printed replicas: a Mayan funerary mask, the Chicén Itzá pyramid and glyphs of the Mayan script that were combined to write words or sentences. Each team would choose one (or more) of the proposed objects and use the information on the sheet as a starting point to envision how they would like to create an interactive exhibition by augmenting the object with digital technology. Participants had internet access and could also look for additional information about the physical objects so that they were not constrained by the design probes provided in the experiment. We introduced the design task before participants had experimented with the toolkit. In this way, their design would not have been biased and limited by the current toolkit functionality. Instead, participants were free to come up with design ideas independently of the technical implementation and then verify whether the toolkit offers the means to materialize their concepts or not. 

### 4.2. Exploratory Workshop

**Figure 11:**
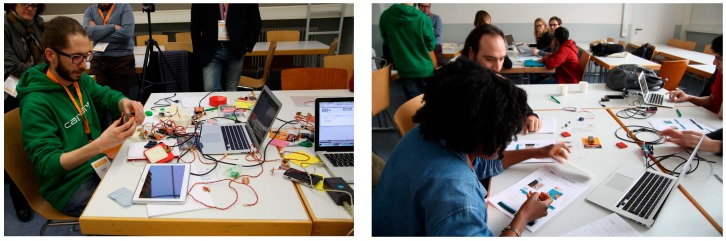
Hands-on experiences with the ECCE toolkit at the TEI studio. On the **left**, one of the organizers preparing a demonstration of the toolkit. On the **right**, participants going through the Arduino/Tinkerkit tutorial.

We ran a one-day hands-on studio [14] at the Tangible, Embodied and Embedded Interaction (TEI) conference (Figure 11). The studio accommodated 8 participants (3 females, age range from 22 to 42, M = 28, SD = 6.12): 3 master students in Interaction Design, 2 industrial designers, 2 Ph.D. students in Computer Science and one Interaction Design researcher. Half of the participants already had programming experience with Arduino, but none of them had used the Tinkerkit platform before. The rest of the participants were new to the subject and they did not have any previous programming experience. 

The workshop adhered to the following schedule, with two twenty-minutes stops after step 2 and step 4:

1. *Introduction (one hour).* Participants were given an introduction to the studio objectives, the concept of device ecosystems, opportunities and challenges. The explanation focused on the integration of digital material with physical objects for the creation of digitally-augmented exhibits. At the end of the introduction, participants were arranged into two groups of four members, balancing each group expertise according to the participants’ profile (e.g., two tech-savvy members per group).
2. *Technology (one hour).* Participants were introduced to Arduino and TinkerKit. With a step-by-step tutorial we guided participants into learning the basics of Arduino programming and the Tinkerkit environment. The tutorial covered the following subjects: (1) project setup; (2) Arduino and Tinkerkit IDE and coding; (3) sensors and actuators; (4) serial connection; (5) servomotors. Each group was given an Arduino and a TinkerKit starter pack to follow and reproduce practical examples.
3. *Design task (90 min).* The use case of a Mayan exhibit was introduced. An example of how a device ecosystem could be developed to enhance the user experience of the exhibit was also provided as reference for the participants. Then, each group chose at least one object and envisioned the interaction they wanted to implement. Participants were free to use whatever technique they might like for conceptualizing the interaction, such as sketching, storyboarding, paper-based mockups or stop motion animations.
4. *ECCE toolkit (30 min).* Participants were introduced to the ECCE toolkit. Again, participants were guided into the basics of the toolkit by means of a step-by-step tutorial. Each step focused on one of the phases defined by the toolkit. Participants learnt: (1) how to create/edit devices; (2) how to to define interaction rules; and (3) how to run and test the resulting device ecosystem.
5. *Implementation task (90 min).* Each group was asked to implement at least one of their design concepts by using the ECCE toolkit and the hardware at their disposal: TinkerKit and Arduino boards with a number of sensors and actuators, Android tablets and smartphones, pico-projectors and laptops.
6. *Presentations and conclusions (30 min).* To conclude, participants presented their designs to the other groups. Presentations focused on the envisioned interaction and implementation issues. The feedback from each group were used as seeds for a wrap-up discussion on the benefits and drawbacks of the ECCE toolkit as well as for closing the studio and highlight directions for future work.

We organized participants in groups because we wanted to observe how the toolkit would be used by a design team with different expertise and background. During the workshops, we did not offer help in the design and implementation tasks. 

### 4.3. Implemented Designs in the Exploratory Workshop

We tested the robustness of the software in a real scenario and confirmed that there were no serious software issues that would incapacitate the toolkit use outside the laboratory and in semi-in-the-wild setting. The exploratory study also showed that the current implementation of the toolkit is mature enough to provide insights about its usefulness for end users.

**Figure 12:**
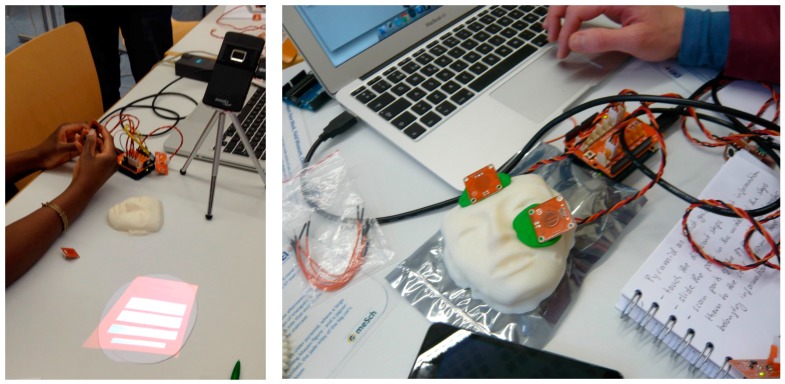
Physical buttons (**left**) and touch sensors (**right**) are used to interact with a 3D replica of a funerary mask.

Figure 12 shows examples of two implemented designs, one for each group using the same artifact (a 3D replica of a funerary mask). The first group wanted to develop a console with physical buttons that would display/hide projected content on the mask (on the left). To this end, the group used a pico-projector to overlay the additional content on the mask—they created a custom device as explained in Section 3.5—and an Arduino with Tinkerkit and four buttons, each one to display/hide the content accordingly. While the first group envisioned that the users would not be able to interact with the artifact directly (they considered the replica as if it were the real artifact), the second group decided to use the 3D replica as a prop to interact with the real artifact. Participants attached touch sensors on the replica (on the right), which would activate digital content. They decided to prototype the interface on the laptop and they envisioned the content to be displayed in the room or directly on the mask. 

### 4.4. Second Workshop

After the TEI studio, we ran a second four-hour workshop with 16 participants (nine females, age range from 22 to 40, M = 29.5, SD = 4.95): 3 HCI/Interaction Design researchers, six PhD students in Computer Science and seven Master students in Computer Science. We ran two different sessions (nine and seven participants) at the University Carlos III de Madrid. Participants were grouped in teams of three and four people according to their technical skills. Participants’ technical skills were assessed with a 7-point scale pre-test questionnaire: they were familiar with ubiquitous computing issues (M = 3.5, SD = 1.8) and have direct experience with the design and implementation of interactive systems (M = 4.8, SD = 1.2), even if most of them (10 out 16) have had no previous experience with the physical computing, electronics and programming micro-controllers. The workshop followed the same design and protocol of the TEI 2014 studio. Additionally, each participant was given a USE questionnaire [46] which assesses the preliminary usefulness, ease of use, ease of learning and satisfaction. Participants’ answers were collected on a 5 points Likert scale.

### 4.5. Designs Implemented during the Second Workshop

**Figure 13:**
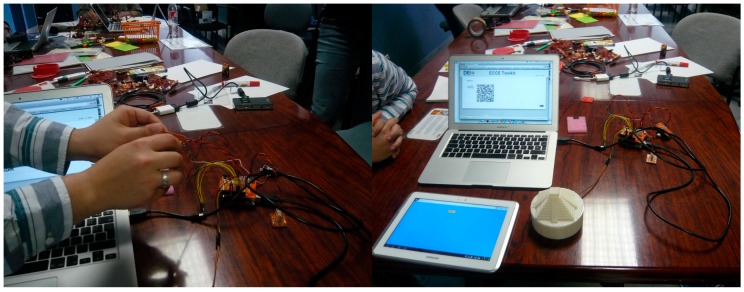
The implementation of a design idea with ECCE during the second workshop: (**left**) a participant connecting the sensors and, (**right**) the final implementation.

All groups provided an implementation of at least one design idea at the end of the workshop. The final solutions implemented simple interactions and were quite similar. This is ascribable to several factors: the limitations of the laboratory setup (time constraints), the fact that participants used the toolkit for the very first time and also the set of sensors and actuators that are supported by the current implementation. Most of the implemented prototypes made use of proximity sensors to show or hide some digital content depending on the distance of a visitor from an exhibition piece. The augmented information, textual or pictorial, was in generally displayed directly over the piece by means of pico-projectors. Three groups exploited the tablets. One of them placed a tablet near an exhibition piece and used it to present interactive digital information (see Figure 13). The tablet offers the advantage, if compared to projected content, to allow visitors to interact with digital content via multi-touch input. For instance, the group used a distance sensor to perceive the presence of a visitor and show a graphical interface on the tablet. The graphical interface provided touch areas that displayed multimedia content related to the piece. Two other groups used the tablets as personal mobile devices to display and interact with digital information. 

Two groups explored other kinds of touch-based interactions. For instance, they used touch sensors for implementing interactive areas on a replica of a piece in the same way other group did in the exploratory workshop: if a visitor touches the interactive area some content is displayed. Only one group explored more complex interactions. They used a rotary potentiometer and a high power LED to simulate the sun lighting up the Chicén Itzá pyramid at different hours of the day. They also connected the rotary potentiometer with a servomotor and put the 3D representation of the pyramid on a platform attached to the servomotor. In this way, they were able to simulate the shadows casted by light, depending of the hour. Another group used the Mayan glyphs and implemented a game-based application that would unveil multimedia content, provided the glyphs are ordered in the correct sequence. To this end, they had to extend the functionality of the toolkit as shown in Section 3.6. One of the groups also designed motion gestures interaction devices to activate events and retrieve content: they envisioned 3D replicas of other common objects of the Mayan culture (such as daggers, hats or staffs) with embedded motion sensors that can enable the detection of gestures like shaking, swiping, etc. This scenario highlighted one of the current limitations of the toolkit: motion detection is only supported to a basic level (e.g., acceleration on the three axis, tilt or orientation) and the data is not exploited to enable more complex interactions. This is one feature that will be considered for the future development of the toolkit, since gestural interaction would increase the breadth of interactive prototypes that can be built. 

### 4.6. Results of the Questionnaire

**Figure 14:**
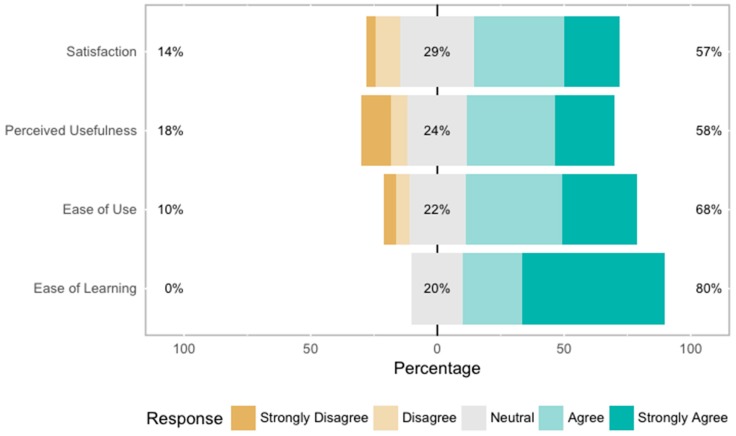
Results of the USE questionnaire according to the four categories: satisfaction, perceived usefulness, ease of use and ease of learning.

Figure 14 shows the results of the USE questionnaire according to the four categories. The questionnaire points out a general positive acceptance of the toolkit. The one-sample Wilcoxon Signed-rank test shows that Likert scores were significantly different (higher) from a neutral value of 3 for all the criteria of the USE approach: satisfaction (M = 3.554, SD = 1.145, ρ < 0.05, 95% CI [3.5, 4.5]), perceived usefulness (M = 3.455, SD = 1.328, ρ < 0.05, 95% CI [3.0, 4.0]), ease of use (M = 3.767, SD = 1.148, ρ < 0.05, 95% CI [4.0, 4.5]), ease of learning (M = 4.359, SD = 0.804, ρ < 0.05, 95% CI [4.5, 5.0]).

## 5. Overall Findings

All the participants of the two workshops were able to successfully use the toolkit in the design process and implement their designs. They appreciated the toolkit support in arranging the hardware medium and abstracting from the technical implementation. After their experience with basic Arduino tasks, participants found the web interface of ECCE as well as the fact that they were not required to employ textual programming to be a major improvement. We, in fact, confirmed findings from the study of Booth’s et al. [19] and found that participants, when using the Arduino IDE, struggled the most in correctly reading data from sensors and that, most of the time, participants were not sure if the source of a problem was hardware or software. ECCE offers a *path of least resistance* [44] that supports user in both circuit and program construction who, thus, can avoid fatal problem such as miswirings or not using sensor readings correctly.

Participants learned quickly how to use the toolkit even if they feel they will need time to get really proficient with it and be able to develop prototypes that involve more complex interactions. They also recognized that the toolkit provides features that are easy to remember: this is an important factor for the adoption of the toolkit by casual or non-technical end users. However, due to the limited range of the experiment, it is not really possible to evaluate to what extent the memorability of the toolkit still holds after longer periods of not use. 

The prototypes developed during the workshops provide tentative evidence that ECCE enables end users to be involved in the design process. To the best of our knowledge, there are no other tools that support the integration of physical and digital components in the same application without making use of textual programming. Given the lack of a baseline, the user study can only show that, by providing an easy entry point for end users with no programming expertise, ECCE allowed multi-disciplinary design teams to implement interactive prototypes in a limited amount of time. ECCE showed potential to support the design process of physical interfaces. All the groups used the toolkit as a “constructive” tool in the design process; ECCE allowed them to quickly materialize their concept designs and use the prototypes for discussion and iteration. Non tech-savvy users observed that they would not have been able to implement even the simplest interaction in one day without the toolkit, due to the high technical knowledge needed to integrate all the different technologies. They reported that they would not even have tried to design and implement such interactions among heterogeneous devices, scared by the technological barriers. This is resonant with findings that toolkits for novel and unfamiliar applications foster creative design [9]. From the other side, participants with high technical expertise felt somewhat limited by the high-level abstraction. One group was able to integrate the basic functionality for an RFID reader using the procedure described in Section 3.6 and with guidance from the researchers. However, they considered that the toolkit would benefit from providing different level of support that span from untrained to skilled programmers. In particular, they would have liked the interface to provide a JavaScript text editor to customize interaction rules once defined. They considered that expert users and developers would benefit from the availability of an API that would allow them to directly program the JavaScript code and add custom functions to the auto-generated code. 

The limited time of the workshop did not allow to test thoroughly whether or not the toolkit raises the ceiling. The kind of interactions developed were also constrained by the set of sensors, actuators, interface elements and interaction rules. It would be difficult to assess which of these factors—available functionality/components and time for prototyping—had a greater impact on the variety of outcomes. 

## 6. Conclusions and Future Work

Developing interactive systems that span across heterogeneous devices is complex. In this paper we have presented the ECCE toolkit that aims at supporting this task by providing tools to ease the design and development of sensor-based physical interaction. ECCE advances the state of the art by providing a toolkit that ease both the physical and software construction so that users avoid typical errors of physical computing and can focus on their design without having to struggle with low-level problems. With our toolkit, end users will be able to rapidly build low-cost functional prototypes that combine interactions between real world data from sensors/actuators and digital user interfaces. They will be encouraged to explore different alternatives, thanks to the easiness of modifying the behavior of existing interactive components and/or integrating new components in the design. Overall, participants felt satisfied with the toolkit. They enjoyed using it, which in most cases stimulated exploration through the generation of different prototypes just for the fun of it. 

We plan to develop the toolkit as an open-source project, which would allow a community of users from different backgrounds to use and contribute to the development of the toolkit by adding and sharing new functionality themselves or showing the kinds of projects that have been developed with the toolkit. 

Future development for ECCE and the field of end-user development for physical computing will focus on extending the toolkit functionality in order to seamlessly support different users’ expertise. Providing scripting mechanisms for expert users would increase the user population of ECCE and allow to understand programming strategies for physical computing of both novice and experienced programmers. We also plan to include features that enable to program spatial relations among devices and users, as already supported in previous scripting toolkits such as XDStudio [22] or Proximity Toolkit [47]. We are also investigating [32] how to extend the toolkit in order to define “virtual sensors”, that is, how to include sensor events from virtual worlds. This would enable end users to seamlessly setup cross-reality environments that interweave the physical and the virtual world. 

## Figures and Tables

**Table 1:** Overview of the state of the art according to the challenges addressed by the ECCE toolkit. We used Harvey Balls ideograms to provide a qualitative evaluation, from 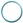 meaning “no support”, to 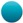 meaning “full support”.

Toolkit	Physical/Digital Interactions	Cross-Device Programming	Build Custom Interactive Objects
Arduino	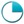	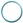	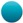
Phidgets	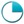	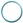	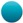
Tinkerkit	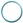	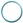	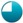
iStuff/iStuff mobile	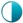	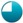	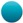
Panelrama	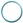	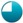	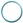
WatchCONNECT	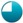	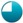	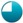
XDStudio	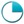	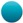	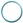
Waive	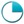	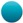	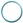
d.tools/Exemplar	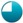	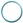	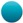
Scratch4Arduino	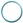	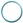	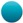
Node-RED	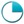	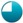	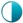
App Inventor	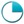	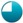	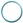

**Table 2:** List of sensors, input devices and actuators currently supported by the ECCE Toolkit.

Sensors	Input Devices	Actuators
Touch	Linear Potentiometer	Micro Servomotor 180°
Light Dependent Resistor (LDR)	Rotary Potentiometer	Micro Servomotor 360°
Force Sensitive Resistor (FSR)	Push Button	Led
GP2Y0A21YK IR Proximity	Toggle Button	Relay
Tilt	Relay	Firgelli PQ12 Micro Linear Actuator
